# Clinical Course of Bio Naive Ulcerative Colitis Patients Five Years After Initiation of Adalimumab in a Nationwide Cohort

**DOI:** 10.1093/crocol/otae046

**Published:** 2024-08-21

**Authors:** Ramaswamy Sundararajan, Manthankumar Patel, Janak Bahirwani, Chinmay Trivedi, Nadim Mahmud, Nabeel Khan

**Affiliations:** Department of Gastroenterology, Corporal Michael J Crescenz VA Medical Center, Philadelphia, PA, USA; Department of Gastroenterology, Corporal Michael J Crescenz VA Medical Center, Philadelphia, PA, USA; Department of Gastroenterology, St. Luke’s University Health Network, Bethlehem, PA, USA; Department of Internal Medicine, Hackensack Meridian Health – Palisades Medical Center, North Bergen, NJ, USA; Department of Gastroenterology, Corporal Michael J Crescenz VA Medical Center, Philadelphia, PA, USA; Division of Gastroenterology, University of Pennsylvania, Perelman School of Medicine, Philadelphia, PA, USA; Department of Gastroenterology, Corporal Michael J Crescenz VA Medical Center, Philadelphia, PA, USA; Division of Gastroenterology, University of Pennsylvania, Perelman School of Medicine, Philadelphia, PA, USA

**Keywords:** Ulcerative Colitis, adalimumab, bio naïve, endoscopic remission

## Abstract

**Background:**

There is limited data on the long-term clinical outcomes of bio-naïve ulcerative colitis (UC) patients who are initiated on adalimumab (ADA). Our study aims to evaluate the clinical course of a nationwide cohort of bio naïve UC patients who were started on ADA, and then followed for 5 years after initiation of the drug.

**Methods:**

We conducted a retrospective cohort study using the US Veteran Affairs Healthcare System (VAHS). Bio naïve UC patients were followed for 5 years after initiation of ADA. The primary outcome was to determine the time to discontinuation of ADA and if patients achieved endoscopic remission by the end of follow-up.

**Results:**

A total of 387 patients were included among whom 193 (49.87%) had pancolitis. The highest rate of ADA discontinuation was within the first year, with the elderly having a higher rate of discontinuation (HR 1.67, 95% CI: 1.14–2.45) and those on concomitant immunomodulators having a lower rate of discontinuation (HR 0.70, 95% CI: 0.48–1.03). In total, 125 (32.30%) patients remained on ADA at the end of their maximum follow-up. 54 (43.90%) achieved endoscopic remission.

**Conclusion:**

Among bio-naive UC patients who were started on ADA, a third were still on the drug at the end of 5 years and half had endoscopic remission. The rate of discontinuation was highest within the first year of initiation, but patients continued to stop the drug over the course of follow-up.

## Introduction

Adalimumab (ADA) is a human IgG1 monoclonal antibody directed against the Tumor necrosis factor (TNF) α receptor that was approved by the FDA in 2012 for the treatment of moderate to severe ulcerative colitis (UC).**^1^** Being a self-injectable, it has been extensively used in the management of UC, and recently biosimilars of adalimumab have also been approved by the FDA**^2,3^** which may further increase the utilization of the drug. In addition to anti-TNF agents, over the last decade, drugs with different mechanisms of action have been approved for the management of UC, including vedolizumab (anti-integrin inhibitor),**^4^** ustekinumab (IL-23 inhibitor),**^5^** ozanimod (Sphingosine-1-phosphate receptor modulator),**^6^** and tofacitinib and upadacitinib (Janus kinase inhibitors)**^7,8^** giving providers multiple options with which to treat the disease.

While ADA has been approved for the management of UC for more than a decade, there is limited data on the long-term efficacy of the drug. Most of the studies have the limited number of patients and variable periods of follow-up. The most comprehensive information can be attained from the long-term follow-up of patients who were enrolled in the regulatory trials and were then followed in open-label extension studies. While clinical trials are essential for regulatory approval, they have placebo arms and are not representative of the patient population encountered in clinical practice due to strict inclusion and exclusion criteria.**^9^**

Considering the above, it is imperative to have real-world data on long-term outcomes after starting ADA, especially as there are multiple medicines currently available for the management of UC while keeping surgical options in perspective. To address this knowledge gap, we conducted a retrospective study using a nationwide cohort of patients with UC followed by the Veteran Health Administration.**^10^** Our aim was to evaluate the clinical course of bio-naïve UC patients who were started on ADA, and then followed for 5 years after the initiation of the drug.

## Materials and Methods

### Study Population

We conducted a retrospective study using a nationwide cohort from the US Veteran Affairs Healthcare System (VAHS) database. We identified patients with UC based on a previously validated algorithm.**^11^** In this cohort we identified patients who had no previous exposure to any anti-TNF or other biologic agents prior to the start of therapy with ADA and who had been followed in the VA for at least 1 year prior to the start of ADA. The charts of all these patients were then individually reviewed to ensure that they all were (1) diagnosed with UC which was confirmed by an endoscopic evaluation prior to ADA administration (baseline colonoscopy), (2) started on ADA, and (3) biologically naïve prior to ADA initiation. This study involved patients newly initiated on ADA before April 2017 to allow for 5 years of follow-up.

### Exposures

Baseline characteristics of patients including age, sex, race, BMI, smoking, baseline colonoscopy findings, prior medication exposure (corticosteroids and immunomodulators), and concurrent medications were assessed. The colonoscopy findings were recorded using criteria defined by Montreal classification extent (0, 1, 2, and 3).**^12^** Baseline laboratory parameters such as albumin, hemoglobin, white blood cell count, and platelet count were obtained in the three months prior to ADA initiation to reflect baseline health status. Baseline ESR and CRP were not collected as on a sample of 100 patients only 37 % had CRP/ESR tested.

### Outcomes

The primary outcome was time to discontinuation of ADA for reasons other than clinical remission. To ascertain this, charts were manually reviewed to determine dates of ADA discontinuation and reasons for discontinuation. Secondary outcomes included determining the clinical course of all the patients who started ADA. This was determined by the medications used to treat the disease over this time period and also whether they attained endoscopic remission. Endoscopic remission was defined as no evidence of inflammation in the colonoscopy report or an endoscopic Mayo score of zero on the last colonoscopy during the 5 years of follow-up.

### Statistical Analysis

To evaluate the cumulative incidence of ADA discontinuation we utilized Kaplan–Meier analysis and plotted failure curves. Observations were right-censored at maximum follow-up or death up to a limit of 60 months. Point estimates with 95% confidence intervals were provided at 6, 12, 24, 36, 48, and 60 months. To identify risk factors associated with ADA discontinuation we used Cox regression analysis. Based on the appearance of Kaplan–Meier failure curves where the rate of failure was notably higher within the first year of follow-up, we focused this analysis on a 12-month time horizon. Given the small cohort sample size and exploratory nature of this analysis we used an alpha threshold of 10% to determine statistical significance and focused on univariable analysis. The following variables were evaluated in models: Age, sex, race, smoking status, BMI, baseline labs, baseline extent of disease, ADA initiation with glucocorticoids, ADA initiation with immunomodulators, and time from UC diagnosis to ADA initiation.

## Ethical Considerations

This project received Institutional Review Board approval from Corporal Michael J. Crescenz Veterans Affairs Medical Center.

## Results

### Baseline Characteristics

A total of 387 patients were included in the study. Most patients were male (91.99%) and White (84.24%), with a mean age of 53.76 years. Baseline colonoscopy showed that close to half of the patients had pancolitis (49.87%). Before starting ADA, most patients had received corticosteroids (86.56%), and over half of them (68.22%) were on steroids concomitantly, at the time of initiation of ADA. Concomitant TP was used by less than half the patients (41.34%; Table 1).

**Table 1.** Baseline characteristics.

		*n* = 387
Male sex, *n* (%)		356 (91.99%)
Age, mean (SD)		53.76 (15.71)
Race	White	326 (84.24%)
	African american	34 (8.79%)
	Not known	21 (5.43%)
	Others	6 (1.55%)
Baseline colonoscopy, extent	1	19 (4.91%)
	2	175 (45.22%)
	3	193 (49.87%)
Albumin, mean (SD)^a^		3.80 (0.56)
Hemoglobin, mean (SD)^b^		13.25 (1.93)
White blood cell count, mean (SD)^c^		8.51 (3.19)
Platelet count, mean (SD)^d^		295.76 (112.65)
BMI, mean (SD)		29.66 (5.72)
Smoking	Never smoker	146 (37.73%)
	Former smoker	129 (33.33%)
	Current smoker	60 (15.50%)
	Unknown	52 (13.44%)
Prior steroid exposure		335 (86.56%)
Prior immunomodulator exposure		203 (52.45%)
Concomitant steroid exposure		264 (68.22%)
Concomitant immunomodulator exposure		160 (41.34%)

Data are presented as mean (SD) for continuous measures, and *n* (%) for categorical measures.

^a^Albumin: 74 out of 387 patients did not have their albumin levels measured at baseline.

^b^Hemoglobin: 40 out of 387 patients did not have their hemoglobin levels measured at baseline.

^c^White blood cell count: 40 out of 387 patients did not have their white blood cell counts measured at baseline.

^d^Platelet count: 40 out of 387 patients did not have their platelet counts measured at baseline.

From the total cohort, 125 patients remained on ADA, at the end of their maximum follow-up period, with 118 patients remaining on ADA at the end of 5 years. 7 patients remained on ADA at their last GI evaluation but did not reach the follow-up period of 5 years. They were either lost to follow-up or left VA GI care Among this group, 41 patients were on a weekly regimen and 84 were on a biweekly regimen at the end of their maximum follow-up. Of these, 54 (43.90%) were in endoscopic remission (Table 2). During follow-up, 60 patients’ dose frequency was transitioned from biweekly to weekly administration.

**Table 2.** Patients who continued adalimumab at the end of follow-up.

Characteristics		*n* = 125^a^^,^^b^	
Dose, *n* (%)	Weekly	41	32.80%
	Biweekly	84	67.20%
Concomitant medications, n (%)	5-ASA	32	25.60%
	Azathioprine	20	16.00%
	Methotrexate	1	0.80%
	Steroids	1	0.80%
	No drug	71	56.80%
Endoscopic remission, *n* (%)		*n* = 123^c^	
		54	43.90%

^a^This includes 118 patients who remained on adalimumab at the end of 5 years as well as 7 patients who were on the drug at their last GI evaluation but did not reach the maximum follow-up period, since they either left the VA or were lost to follow-up.

^b^1 patients out of 126 died during the follow-up period.

^c^2 patients did not have endoscopic evaluation in the 5 years after adalimumab initiation.

### Variables Associated With Adalimumab Discontinuation

**Figure 1.**
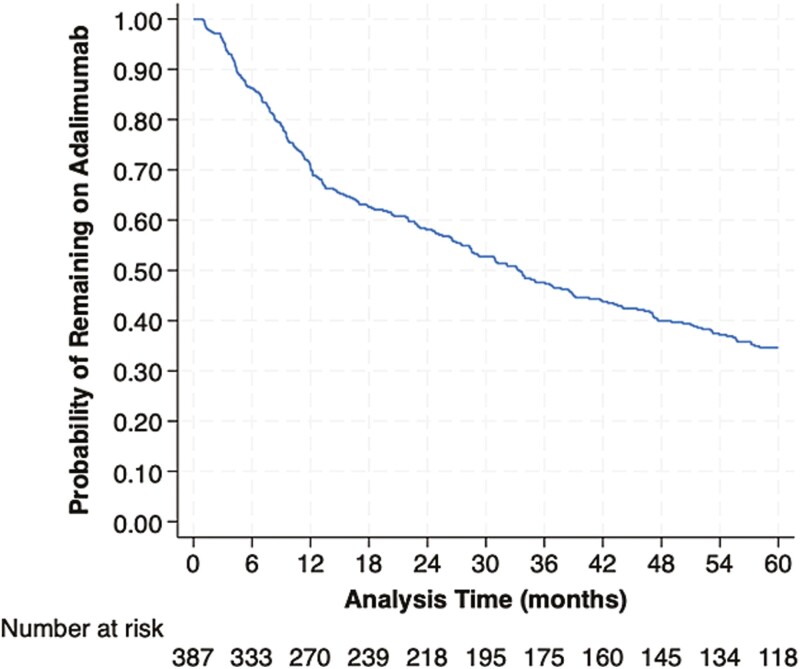
Cumulative incidence of Humira discontinuation in months

**Figure 2.**
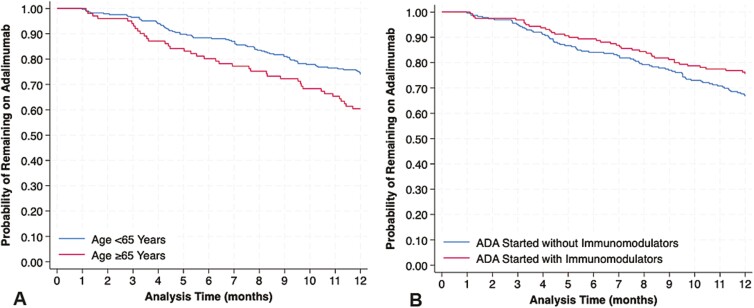
Association between (A) age category and (B) concomitant immunomodulator use and adalimumab discontinuation through 12 months of follow-up

Over median follow-up of 30.9 months (IQR 10.1, 60.0), a total of 247 patients discontinued ADA for reasons other than clinical remission. The cumulative incidence of ADA discontinuation is shown in Figure 1, and cumulative incidences at specific timepoints are shown in Table 3. At 12 months, the cumulative incidence of ADA discontinuation was 29.6%, and through 60 months it was 65.4%. In univariable Cox regression analysis focused on 1-year outcomes, only age category (≥65 vs. <65 years) and ADA initiation with immunomodulators were significant at the 10% alpha level. Patients aged ≥65 years had a 67% increased hazard of ADA discontinuation relative to patients aged <65 years (HR 1.67, 95% CI: 1.14–2.45; Figure 2A), and patients initiated on concomitant immunomodulators had a 30% lower hazard of ADA discontinuation relative to those not initiated on immunomodulators (HR 0.70, 95% CI: 0.48–1.03; Figure 2B). In a multivariable model adjusting for sex, race, and smoking status, patients aged ≥65 years had a 1.70-fold higher hazard of ADA discontinuation relative to aged <65 years (1.70, 95% CI: 1.13–2.56, *P* = .01); patients initiated on concomitant immunomodulators had a 29% reduced hazard of ADA discontinuation (HR 0.71, 95% CI: 0.48–1.05, *P* = .09).

**Table 3.** Cumulative incidence of ADA discontinuation at specified timepoints.

Timepoint (months)	Cumulative incidence of ADA discontinuation	95% confidence interval
6	0.137	(0.107–0.176)
12	0.296	(0.253–0.344)
24	0.419	(0.371–0.470)
36	0.524	(0.475–0.575)
48	0.601	(0.551–0.650)
60	0.654	(0.605–0.702)

### Characteristics of Patients Who Discontinued Adalimumab

Among the patients that discontinued ADA, at the end of 5 years of follow-up, 51 (13.18%) patients underwent colectomy for disease exacerbation.132 patients switched from ADA to other anti-TNF’s or biologics. Of these, 96 patients switched from ADA once. 36 patients switched more than one agent after ADA. Overall, 65 (48.87%) patients were on vedolizumab and 45 (33.83%) were on infliximab. A total of 55 (41.98%) patients were in endoscopic remission (Table 4). Thirty patients were on 5-ASA among whom 10 (34.48%) were in endoscopic remission. Twenty-six patients were on no medications for UC. Of these, 9 (37.50%) were in endoscopic remission. Twelve patients were on thiopurines at the end of follow-up, of whom 7 (63.64%) were in endoscopic remission (Table 5). Over the course of follow-up, 83 patients who discontinued ADA had a dosing frequency change from biweekly to weekly dose. Among these, 13 patients underwent colectomy. The remaining 70 patients transitioned to alternative therapies (Anti-TNF/biologics—59, 5-ASA—3, Thiopurines—1, and no medications—11). One patient stopped ADA on account of developing chronic myeloid leukemia and switched to vedolizumab.

**Table 4.** Patients on other anti-TNF or biologics at the end of follow-up.

Characteristics		Single switch (*n* = 96^b^)	More than 1 switch (*n* = 36)
Anti-TNF/biologics alone or with 5-ASA, *n* (%)	Certolizumab	3 (3.13%)	2 (5.56%)
	Golimumab	1 (1.04%)	0 (0.00%)
	Infliximab	29 (30.21%)	4 (11.11%)
	Vedolizumab	41 (42.71%)	15 (41.67%)
	Ustekinumab	3 (3.13%)	7 (19.44%)
	Tofacitinib	1 (1.04%)	2 (5.56%)
Anti-TNF/biologics + thiopurines/methotrexate, *n* (%)	Golimumab + Azathioprine	2 (2.08%)	0 (0.00%)
	Infliximab + Azathioprine	9 (9.38%)	0 (0.00%)
	Vedolizumab + Azathioprine	3 (3.13%)	4 (11.11%)
	Ustekinumab + Azathioprine	1 (1.04%)	0 (0.00%)
	Infliximab + Methotrexate	2 (2.08%)	1 (2.78%)
	Vedolizumab + Methotrexate	1 (1.04%)	1 (2.78%)
			*n* = 35^a^
Endoscopic remission, *n* (%)		47 (48.96%)	8 (22.86%)

^a^1 patient out of 36 did not have endoscopic evaluation in the 5 years after adalimumab initiation.

^b^1 patients out of 97 died during the follow-up period and were not included in this table.

**Table 5.** Patients on 5-ASA versus no medications for ulcerative colitis versus patients on thiopurines at the end of follow-up.

	Patients on 5-ASA, *n* = 29^a^		Patients on no meds, *n* = 24^b^		Patients on thiopurines, *n* = 11^c^	
Endoscopic remission, n (%)	10	34.48%	9	37.50%	7	63.64%

^a^4 patients out of 34 died during the follow-up period, and 1 did not have endoscopic evaluation in the 5 years after adalimumab initiation.

^b^2 patients out of 26 did not have endoscopic evaluation in the 5 years after adalimumab initiation.

^c^1 patients out of 13 died during the follow-up period and 1 did not have endoscopic evaluation in the 5 years after adalimumab initiation.

## Discussion

In a nationwide cohort of bio-naïve patients with UC who were initiated on adalimumab (ADA) therapy, approximately one-third of the patients remained on ADA at the end of 5 years. Nearly half of those who remained on the drug attained endoscopic remission. The highest rate of discontinuation was within the first year, and individuals aged over 65 demonstrated a higher likelihood of discontinuation during this initial period while concomitant use of immunomodulators was associated with a decreased risk of discontinuation.

Studies to date that have evaluated the long-term clinical course of patients starting ADA, are limited by methodology and variability in follow-up periods. They had median follow-up periods of less than 18 months, included a limited number of bio-naïve patients (ranging from 13% to 87%), and reported variable remission rates (ranging from 11% to 43%). They also had varying rates of ADA discontinuation (ranging from 13% to 70%), attributed to the disparities in follow-up duration and small sample sizes.**^13–17^** The most comprehensive data on longer-term follow-up is derived from extension studies of the placebo-controlled regulatory trials. Suzuki et al in their open-label extension trial of 190 bio-naïve patients out of the 274 who initially started the study, reported that at the end of 4 years, 125 were still on the drug and 30.5% had endoscopic remission.**^18^** The ULTRA 3 maintenance trial evaluated 588 out of the 1094 patients who were enrolled in ULTRA 1 and 2 and they found that at the end of 3 years, 360 patients were still on ADA.**^19^** The long-term outcomes in these trials are difficult to interpret as in the initial period, different dosages and placebo were used and endoscopic remission was defined as endoscopic score of 1 or less without central reading. Furthermore, randomized controlled trials have strict inclusion and exclusion criteria which can limit external validity, and it has been shown that only a quarter of UC patients are eligible to participate in clinical trials.**^9^** These limitations highlight the importance of real-world data in evaluating the clinical efficacy of drugs.

The salient finding of our study was that a third of all bio-naive UC patients who started ADA were on the drug at the end of 5 years. The highest rate of discontinuation was observed in the first year. Interestingly, the rate of discontinuation was much higher in the older population. This is a novel finding, and we were probably able to elicit this considering the large number of elderly patients in our cohort who are often excluded from regulatory trials. A possible explanation for this could be the immune remodeling that occurs with age. Aging results in patients’ B and T cells immunosenescence and alterations in mucosal immunity. Some of these changes are decreased MALT cell frequency, attenuation of the size and number of Peyer’s patches, decreased isolated lymphoid follicle development and immunoglobulin production, decreased epithelial barrier function, and decreased intestinal antigen-specific IgA antibody responses. This may impact the efficacy of ADA among the elderly.**^20^**

The rate of discontinuation was lower in the following 2 years. Approximately 10 % of the cohort stopped the drug each year, followed by 5 % over the subsequent 2 years. These findings indicate that the utilization of the ADA continues to wane over time. Of those who continued the drug, less than half achieved endoscopic remission and at the end of 5 years, 1 in 7 bio-naive UC patients who started ADA were on the drug and had endoscopic remission. This information is vital to prescribers and patients, as they initiate ADA, especially considering other pharmacological options while also keeping the surgical option in consideration.

We also followed the clinical history of patients who stopped ADA. Approximately 13.2 % of patients underwent colectomy for disease exacerbation. While the overall rate of colectomy has decreased since the introduction of anti-TNF agents, 1 in 7 patients still undergo a colectomy.**^21,22^** Approximately one-third of patients switched to other anti-TNF therapies or biologics, like infliximab, vedolizumab, ustekinumab, etc. with the majority having a single switch. Vedolizumab and Infliximab were the most common drugs used at the time of switching. However, despite the switches, a little under half the patients had endoscopic remission at the end of follow-up. This information is also of great prognostic value while considering a switch to another agent. A smaller number of patients were on TP alone, mesalamine alone, or on no therapy. Cumulatively 40.63 % of this group had endoscopic remission. There was also a small subset of patients who stopped ADA (3.62%) because they were in remission.

A major strength of our study is the granular chart review done using the Nationwide Veteran Affairs Health System (VAHS) which caters to over 9 million veterans annually.**^23^** A comprehensive review of individual charts of patients with their endoscopic reports, progress notes, and review of medications enabled us to ascertain all relevant information with precision. As patients tend to maintain their care in the VA, we had excellent longitudinal follow-up in a real-world setting. These factors enabled us to follow the largest cohort of bio naïve patients over a long period of time. However, this study is not without its limitations. Firstly, since it is a retrospective study, it is subject to the inherent limitations of retrospective studies. Patients were not evaluated at fixed intervals and all elements were not recorded. To address this issue, we individually reviewed each chart and evaluated each progress note as well as endoscopy over the entire follow-up period. Biomarkers such as CRP, ESR, and calprotectin were rarely recorded which precluded us from evaluating them. Most of the patients were male and older which may limit the external validity of the study. However, to date, no study has shown any impact of sex on the clinical outcomes of patients with UC.

## Conclusion

The management of UC has undergone a transformation in the last decade with the introduction of multiple new medications with different mechanisms of action. Furthermore, biosimilars of anti-TNF agents have led to increased utilization of these drugs. Despite all these advancements a significant number of patients are still undergoing surgery for management of the disease. Our study found that 1 in 7 bio-naive UC patients who initiated ADA were still on the drug at the end of 5 years and had endoscopic remission. The highest rate of discontinuation was in the first year, but it continued in the subsequent years. This information will be very valuable to patients and physicians as they decide upon a treatment plan for moderate to severely active UC patients.

## Data Availability

The data for this manuscript cannot be made available in accordance with the HIPAA rules. However, de-identified data (without patient name and SSN), can be made available upon reasonable request.

## Author Approval

All the authors have reviewed and approved the final version of this article.
